# Clinical challenges in interpreting multiple pathogenic mutations in single patients

**DOI:** 10.1186/s13053-021-00172-3

**Published:** 2021-02-04

**Authors:** Christa Slaught, Elizabeth G. Berry, Lindsay Bacik, Alison H. Skalet, George Anadiotis, Therese Tuohy, Sancy A. Leachman

**Affiliations:** 1 Department of Dermatology, Oregon Health & Science University, 3303 SW Bond Ave, Suite 16D, Portland, OR 97239 USA; 2 Department of Dermatology, Penn State Health, Hershey, USA; 3 Department of Ophthalmology, Casey Eye Institute, Oregon Health & Science University, Portland, USA; 4 Knight Cancer Institute, Oregon Health & Science University, Portland, USA; 5 Department of Radiation Medicine, Oregon Health & Science University, Portland, USA; 6 Legacy Cancer Institute, Cancer Genetics Services, Legacy Health Systems, Portland, USA

**Keywords:** Melanoma, Genetics, Compound heterozygosity, BAP-1, MSH6, RECQL4

## Abstract

**Background:**

In the past two decades, genetic testing for cancer risk assessment has entered mainstream clinical practice due to the availability of low-cost panels of multiple cancer-associated genes. However, the clinical value of multiple-gene panels for cancer susceptibility is not well established, especially in cases where panel testing identifies more than one pathogenic variant. The risk for specific malignancies as a result of a mutated gene is complex and likely influenced by superimposed modifier variants and/or environmental effects. Recent data suggests that the combination of multiple pathogenic variants may be fewer than reported by chance, suggesting that some mutation combinations may be detrimental. Management of patients with “incidentally” discovered mutations can be particularly challenging, especially when established guidelines call for radical procedures (e.g. total gastrectomy in CDH1) in patients and families without a classic clinical history concerning for that cancer predisposition syndrome.

**Case presentation:**

We present two cases, one of an individual and one of a family, with multiple pathogenic mutations detected by multi-gene panel testing to highlight challenges practitioners face in counseling patients about pathogenic variants and determining preventive and therapeutic interventions.

**Conclusions:**

Ongoing investigation is needed to improve our understanding of inherited susceptibility to disease in general and cancer predisposition syndromes, as this information has the potential to lead to the development of more precise and patient-specific counseling and surveillance strategies. The real-world adoption of new or improved technologies into clinical practice frequently requires medical decision-making in the absence of established understanding of gene-gene interactions. In the meantime, practitioners must be prepared to apply a rationale based on currently available knowledge to clinical decision-making. Current practice is evolving to rely heavily on clinical concordance with personal and family history in making specific therapeutic decisions.

**Supplementary Information:**

The online version contains supplementary material available at 10.1186/s13053-021-00172-3.

## Background

Use of multi-gene panel testing (MGPT) with next-generation sequencing (NGS) for the diagnosis of hereditary cancer predisposition has increased significantly over recent years [1]. Due to the rise in utilization and affordability of MGPT, more individuals are being identified with inherited germline mutations in what are believed to be well-described, moderately and highly penetrant genes. The phenomenon may be rare. A 2018 review of *BRCA1* and *BRCA2* double heterozygotes estimated the frequency of occurrence in the non-Ashkenazi Jewish population of 1:190,000 [2]. Recent evidence presented by one of the larger germline cancer predisposition clinical testing companies (Invitae) showed that ~ 5% of 264 pancreatic cancer patients who tested positive for pathogenic or likely pathogenic mutations in a panel of 84 genes had 2 or more such mutations [3]. Although statistical significance cannot be assessed, the results may be fewer than reported by chance, suggesting that some genetic combinations of mutations may be detrimental.

Often, these patients do not meet established clinical criteria for a known cancer syndrome. Accurate prediction of the functional consequences of identified genetic variants plays a vital role in the field of genetic test reporting. However, the exact effect of genetic mutations on protein function is often not known. Furthermore, the risk for specific malignancies as a result of a mutated gene/protein is complex with the potential for superimposed modifier gene and environmental effects that often remain incompletely understood. We highlight two scenarios with combinations of more than one pathogenic (or likely pathogenic) mutation to illustrate the challenges practitioners face in incorporating genetic testing results into clinical practice (Table 1):

**Table 1:** Classic Presentations of Tumor Predisposition Syndromes and the Molecular and Clinical Characterization of Two Individuals with Multiple Pathogenic Mutations

Table 1. Classic Presentations of Tumor Predisposition Syndromes and the Molecular and Clinical Characterization of Two Individuals with Multiple Pathogenic Mutations										
	**BAP1 Mutation**			**MSH6 Mutation**			**RECQL4 Mutation**			
**Gene Information**	**Gene type:** Nuclear-localized deubiquitinase enzyme Syndrome: BAP-1 Tumor predisposition syndrome Inheritance: AD **Associated malignancies:** Uveal melanoma, mesothelioma, clear cell renal cell carcinoma, atypical Spitz tumors, cutaneous melanoma, basal cell carcinoma **Other Malignancies:** Meningioma, cholangiocarcinoma, lung cancer, breast cancer, ovarian cancer			**Gene type:** Mismatch repair gene **Syndrome:** Lynch Syndrome Inheritance: AD **Associated malignancies:** Colorectal cancer, endometrial cancer, ovarian cancer, urothelial cancer, CNS cancer, GI cancer, pancreatic cancer **Other malignancies:** Breast cancer			**Gene type:** DNA helicase gene Syndromes: Rothmund-Thompson syndrome (RTS), Baller-Gerold syndrome (BGS), RAPADILINO syndrome **Inheritance:** AR **Associated malignancies:** Osteosarcoma, Lymphoma, keratinocyte carcinomas (RTS, BGS)			
	**Variant**	**Pathogenicity**	**Associated Tumors**	**Variant**	**Pathogenicity**	**Associated Tumors**	**Variant**	**Pathogenicity**	**Associated Tumors**	**Other Non-syndromic Cancers**
**Family 1**^**a**^										
**Patient 1**^**b**^	Ex12 c.1185dupA (p.Asp396Argfs^f^2)	Pathogenic	Basal cell carcinoma of face (×2)	Intron 9 c.4002-2A > G (splice acceptor)	Likely pathogenic	Colon cancer (loss of MSH6)	Exon 14 c.2296C > T (p.Gln757^f^)	Pathogenic (hetero-zygous)	Basal cell carcinoma of face (×2)	Hepatocellular carcinoma with recurrence, Lung cancer
**Mother**	*Declined testing*		Breast cancer	*Declined testing*		Breast cancer	*Declined testing*			
**Father**	*Declined testing*			*Declined testing*		Bladder cancer	*Declined Testing*			
**Sister**	*Declined testing*		Breast cancer	*Declined testing*		Breast cancer	*Declined testing*			
**Brother**	*Declined testing*			*Declined testing*			*Declined testing*			Prostate cancer
**Family 2**										
**Patient 2**^**c**^ **(IV-5)**	c.38-1G > A	Likely pathogenic		NEGATIVE						
**Mother**	NEGATIVE			Ex3_9del	Pathogenic	Breast cancer				
**Father**	*Not tested*		Renal cell carcinoma	*Not tested*						
**Sister 1 (IV-3)**	NEGATIVE			NEGATIVE						Non-melanoma skin cancer
**Sister 2**^**d**^ **(IV-4)**	NEGATIVE		Breast cancer (intact MLH1, MSH2, MSH6 and PMS2)	Ex3_9del	Pathogenic	Colon cancer (loss of MSH6**)**, Endometrial cancer				
**Sister 3 (IV-6)**	c.38-1G > A	Likely pathogenic		Ex3_9del	Pathogenic	Precancerous colon polyps				
**Paternal Uncle**	*Not tested*		Cutaneous melanoma	*Not tested*						
**Paternal Cousin**^**e**^	*Not tested*		Ocular melanoma, Renal cell carcinoma	*Not tested*						

*VUS* Variants of unknown significance

^a^All family members for Patient 1 declined genetic testing due to concerns for discrimination by insurance companies

^b^Patient 1: The proband and index case for Family 1. Diagnosed with fibrolamellar hepatocellular carcinoma at age 38 and underwent left hepatic lobectomy (initial staging unknown). At age 56, she was found to have locally recurrent hepatocellular carcinoma, requiring wedge resection. On peri-operative imaging, she was also diagnosed with primary lung cancer and underwent left upper lung lobectomy and mediastinal lymph node resection (well- to moderately-differentiated adenocarcinoma with bronchioloalveolar carcinoma features, stage 2A). She had two basal cell carcinomas (BCCs) of the face at ages 58 and 60 treated with Mohs micrographic surgery. At age 67, she was diagnosed with colon cancer requiring right hemicolectomy (poorly differentiated adenocarcinoma with loss of MSH6 on immunohistochemistry, stage 1)

^c^Patient 2: Proband for family 2

^d^Sister 2 (IV-4 on pedigree 2): The index case for Family 2. At age 54, she underwent right hemicolectomy that revealed a primary colon cancer (moderately differentiated mucinous adenocarcinoma, stage 2). Loss of nuclear expression of *MSH6* suggested a high probability of Lynch syndrome and Ambry Genetics panel testing revealed an *MSH6* EX3-9del. She subsequently underwent a prophylactic TAHBSO, which revealed early endometrial cancer (grade 1, stage IA). At age 55, she was diagnosed with left breast cancer on routine mammography (ER/PR negative, stage 1B). Immunohistochemical studies performed on the breast biopsy tissue demonstrated intact expression of DNA mismatch repair proteins MLH1, MSH2, MSH6 and PMS2, suggesting that her breast cancer was sporadic and not associated with microsatellite instability

^e^Paternal Cousin: Renal cell carcinoma (stage 4)

^f^Malignancies are color-coded according to the patients and mutations they have been associated with (BAP1, BAP1 and MSH6, MSH6, RECQL4, BAP1 and RECQL4)

## Case presentation

Patient 1 is a 70-year-old Caucasian woman who was referred for genetic testing given her extensive personal history of cancer as listed in Table 1. She underwent Invitae Multi-Cancer panel genetic testing (Supplemental Table 1) that detected heterozygous mutations in BRCA1-associated protein 1 (BAP1), MutS Homolog 6 (*MSH6)* and ATP-dependent DNA helicase Q1 (*RECQL4)* genes, all noted to be “pathogenic” or “likely pathogenic.”

Patient 2 underwent genetic testing due to her known familial MSH6 mutation and family history of cancer concerning for germline BAP1 mutation. Ambry genetics site-specific testing revealed only the familial *BAP1* mutation in our patient. However, her youngest sister (IV-6) tested positive for both the *BAP1* and *MSH6* mutations.

## Discussion

### Defining pathogenicity

One important consideration in clinical management is the pathogenicity of the identified gene variants. “Pathogenic” and “likely pathogenic” variants imply that the mutation warrants surveillance according to full high-risk guidelines and qualifies for predictive testing of at-risk relatives [4]. On the other end of the spectrum, “likely non-pathogenic” and “non-pathogenic” variants suggest that the genetic change is a polymorphism or normal variant and is to be treated as if no mutation was detected [4]. Between these categories is a “variant of unknown significance,” where clinical management is decided, case-by-case, based on personal and family history and other risk factors, as further understanding evolves [4, 5].

The reliability of variant classification has improved in recent years with the introduction of variant classification frameworks. In response to the observation that variant classifications could differ between laboratories, the American College of Medical Genetics and Genomics-Association for Molecular Pathology (ACMG-AMP) established guidelines to create a common framework for variant classifications in 2015 [6]. In 2017, “Sherloc” (semiquantitative, hierarchical evidence-based rules for locus interpretation) was developed as a refinement of the ACMG-AMP criteria [7]. All interpreted variants are routinely deposited into ClinVar, for ease of access.

With more widespread use of MGPT, there are also increasing reports of individuals with pathogenic mutations in multiple cancer predisposition genes. A recent review of 55,803 patients tested with a 25-hereditary cancer gene panel, found that 106 (0.19%) had pathogenic or likely pathogenic mutations in two or more genes [8].

Our patients’ mutations were deemed likely pathogenic or pathogenic (Table 1). *MSH6*, *BAP1*, and *RECQL4* mutations are rare individually. To our knowledge, the combinations of genes inherited in these individuals has never been reported.

### Review of mutations

#### BAP1

The *BAP1* gene encodes a nuclear-localized deubiquitinase and plays a pivotal role in epigenetic modification, transcription regulation, and DNA damage response [9]. Germline mutations of *BAP1* are associated with an autosomal dominant, novel cancer syndrome characterized by atypical Spitz tumors (AST), uveal melanoma, mesothelioma (MM), clear cell renal cell carcinoma, cutaneous melanoma (CM), and basal cell carcinoma (BCC) [9–13].

The molecular mechanisms and cellular pathways responsible for leading to these specific tumor types remain unclear [13]. The full spectrum of this syndrome is still being characterized and more recently associated tumors include meningioma, cholangiocarcinoma, hepatocellular carcinoma, lung cancer, and breast/ovarian cancer [13, 14]. Data remains limited regarding accurate estimations of either the lifetime risks or average age of diagnosis for each of these associated cancers [12, 15, 16].

As the full phenotype for mutations in this gene needs additional investigation, numerous screening guidelines have been proposed (Table 2). Screening recommendations support at least annual general physical, ophthalmological and dermatologic examinations [10, 11, 15]. Given the impairment in DNA damage repair associated with *BAP1* mutations, it is suggested that ultrasound and MRI be utilized, when possible, to avoid the radiation associated with CT scans [16]. Other important prevention strategies include photoprotection to minimize ultraviolet radiation exposure (CM/BCC risk), avoidance of arc welding (UM risk) and avoidance of smoking or asbestos exposure (MM risk) [16].

**Table 2:** Malignancy Screening Recommendations by Gene

	Associated Neoplasms	Age^a^	Recommendation
BAP1	Uveal melanoma	11	Annual dilated eye exams by an ocular specialist
	Mesothelioma	–	Annual physical +/− Chest MRI
	Cutaneous melanoma, BCC, AST	20	Annual or biannual full body skin exam
	Clear cell renal cell carcinoma	–	Annual physical Annual abdominal ultrasound Annual urinalysis Abdominal MRI every 2 years
MSH6	Colon cancer	25	Colonoscopy every 1–2 years
	Endometrial cancer		Prophylactic hysterectomy
	Ovarian cancer	30	Prophylactic bilateral salpingo-oophorectomy Prior to prophylactic removal, consider: Annual transvaginal ultrasound Annual CA-125
	Urothelial cancer	30	Consider annual urinalysis
	Gastrointestinal tract	30	Esophagogastroduodenoscopy every 3–5 years
	Pancreatic cancer	–	Consider endoscopic ultrasound
	CNS malignancy		Annual physical exam with neurologic examination
RECQL4 (biallelic) (RTS, BGS, RAPADILINO Syndrome)	Osteosarcoma		Annual physical Prompt skeletal radiographic evaluation when suspected clinically
	Lymphoma		Annual physical Baseline complete blood count with differential
	Keratinocyte carcinomas (RTS and BGS only)		Annual full body skin exam

^a^Age: Start screening at this age or 5 years prior to earliest age of diagnosis in family if younger than those listed here

*RTS* Rothmund-Thompson Syndrome

*BGS* Baller-Gerold Syndrome

#### MSH6

The *MSH6* gene encodes one protein member of a growing number of heteroduplex complexes of proteins that maintain genomic stability by recognizing and correcting single base mismatches and insertion/deletion loops that may arise during replication [4]. Heterozygous germline mutations in any one of several mismatch repair genes are associated with Lynch syndrome, an autosomal dominant cancer-susceptibility disorder previously known as hereditary nonpolyposis colorectal cancer syndrome. Approximately 90% of mutations associated with Lynch syndrome are located in *MLH1*, *MSH2* and *EPCAM* genes, while 10% are located in *MSH6* and *PMS2 (and a very small number in the more recently identified MSH3)* [17]. Carriers of these mutations are at high risk of early-onset colorectal and endometrial cancer as well as tumors of the ovaries, urothelium, CNS and entire gastrointestinal tract, including the pancreas [17].

Multiple national and international professional screening recommendations have been proposed (Table 2) [18]. Although data indicate a mildly increased risk for breast cancer, no specific recommendations have been made for this risk.

#### RECQL4

*RECQL4* is a DNA helicase gene that has been implicated in DNA double stranded break repair, nucleotide excision repair, and base excision repair, important for DNA replication and repair of UV damage [19–22]. This gene may also play a role in telomere maintenance [23]. Homozygous (or compound heterozygous) germline mutations in the *RECQL4 gene* have been associated with Rothmund-Thompson (RTS), Baller-Gerold (BGS), and RAPADILINO syndromes [24]. These syndromes are autosomal recessive conditions associated with biallelic disruption of the helicase gene (usually compound heterozygous or consanguineous homozygous mutations). RTS, BGS, and RAPADILINO have been associated (albeit rarely) with osteosarcoma and lymphoma, and both RTS and BGS are associated with skin cancer, specifically keratinocyte carcinomas [25]. Thus, in the case of biallelic loss of gene function, evidence suggests an association of *RECQL4* with cancer predisposition [23]. The impact of a heterozygous *RECQL4* mutation on cancer predisposition unknown, particularly in the context of simultaneously inherited DNA damage and repair gene mutations like *BAP1* and/or *MSH6.*

Table 2 includes a summary of the screening recommendations for malignancies associated with RECQL4. In addition to regular screening, photoprotection and minimization of ultraviolet radiation may be important. We recommend a conservative approach to UV and other types of radiation, as the inevitable somatic loss of RECQL4 in random heterozygous cells may lead to increased risks for clonal populations of those cells over time. This idea is supported by a recent report that heterozygous germline carriers of pathogenic RECQL4 mutations were over-represented in children with osteosarcoma [26].

### Multiple pathogenic mutations

Compared to phenotype-driven single gene testing, MGPT increases the probability of identifying pathogenic mutations. Studies of large populations of women diagnosed with breast cancer suggest that nearly 8% carry a pathogenic variant that, if detected, would warrant a change in preventive care, such as secondary breast cancer screening incorporating MRI, early colonoscopy or high risk surgery [27]. In addition, as many as 4% of these individuals have germline pathogenic mutations in cancer predisposition genes other than BRCA1 and BRCA2 on MGPT (i.e. CHEK2, PALB2, ATM, NBN, PTEN, etc.) [27, 28]. Nevertheless, the benefit of more comprehensive genetic testing strategies is often debated. Concerns include cost, increased procedure-related morbidity, and the potential for discovery of uninformative or anxiety-provoking results [29].

Another potential consequence of MGPT is the identification of more than one pathogenic mutation in the same individual or identification of pathogenic mutations that do not match the patient’s phenotype. Review of larger cohorts undergoing MGPT for breast, ovarian and general hereditary cancer risk has revealed that approximately 3% of individuals test positive for pathogenic mutations in multiple genes [30]. This carries significance for both the individual and their family members, suggesting that single-gene testing—even if targeted to known familial pathogenic mutations—may miss other significant pathogenic mutations. A review looking specifically at patients undergoing genetic testing for suspected Lynch syndrome based on history of Lynch syndrome-associated cancer and/or polyps, found that MGPT identified high-penetrance mutations in other non-Lynch Syndrome cancer predisposition genes in 5.6% of these individuals, many of which were unexpected based on patients’ histories [31]. In fact, that study found more than one high-penetrance non-Lynch Syndrome gene mutation for every 5 Lynch Syndrome gene mutations identified, revealing that it is not uncommon to have unexpected, clinically useful findings with MGPT in this population [31]. MGPT should be considered to identify missed pathogenic mutations and more accurately inform hereditary cancer risk.

Identifying patients with multiple clinically actionable mutations has important implications for patients and their family members, yet much remains unclear regarding the consequence of carrying more than one pathogenic mutation. Our understanding of how combinations of mutations may interact to alter the ultimate profile of cancers in a patient, or their family, is currently very limited [32–34]. Certain combinations of mutations may be detrimental in that they increase overall risk of malignancy while others may reduce overall cancer risk, relative to inheritance of the single gene mutation alone. In addition, the remainder of any individual’s genomic background and their cumulative exposure history to cancer-predisposing agents superimpose additional effects on the risks conferred by the high-penetrance genes.

Based on this framework and the limited data we have available; a few effects are possible:

1. Detrimental consequence: Some germline cancer susceptibility mutations may interact in an additive or synergistic manner to increase the penetrance of cancers related to other germline cancer susceptibility mutations. There is some precedence for this in the literature. The rs2304277 variant in the *OGG1 glycosidase* gene of the base excision repair pathway has been shown to increase the penetrance of ovarian cancer in patients with *BRCA1/2* mutations [35]. The mutation was found to independently decrease expression of mRNA *OGG1*, thus contributing to further genomic instability via DNA damage and telomere shortening [35]. In addition, co-inheritance of the *cyclin-dependent kinase inhibitor* (*CDKN2A*) gene and variants of the *melanocortin-1 receptor* (*MC1R*) gene that have previously been associated with red hair, fair skin and sensitivity to ultraviolet light (*Arg151Cys*, *Arg160Trp*, and *Asp294His*) have been found to significantly increase melanoma penetrance and decreased the age of onset by 20 years compared to individuals carrying the *CDKN2A* mutation alone [36]. Earlier onset of colorectal cancer has also been reported in an individual with both compound heterozygous MSH6 mutations and an APC missense mutation, diagnosed at age 18 [37].
2. Beneficial consequence: Increased fragility of cells carrying multiple mutations could counterintuitively result in an overall cancer risk that is less than the sum of the individual risks from the inherited germline mutations. There may be a restricted profile of tertiary and subsequent mutations that a cell can handle. This may lead to increased apoptosis, cell death or senescence as somatic mutations accumulate, which could reduce penetrance or improve prognosis of tumors. For example, as outlined above, germline mutations affecting DNA repair genes, such as *MSH6*, have been clearly associated with a high risk of cancer development [17]. However, gene expression profiling of tumor cell lines and specimens of different histologic origin has also shown that overexpression of DNA repair genes is often associated with more aggressive behavior of cancer cells and lower patient disease-free or overall survival, including melanoma [38]. These findings have led to the hypothesis that, while genetic instability is essential for tumor initiation, it may decrease the potential for progression or metastasis [39].
3. Genomic Background and Environmental Exposure: In addition to the consequence that inherited germline mutations may have on each other, the overall penetrance of a cancer predisposition syndrome is also affected by the individuals genomic background and environmental exposures, such as ultraviolet radiation. For example, protective modifier genes may exist that mitigate the risks conferred by high-penetrance genes. A recent analysis of 589,306 genomes by Friend et al. identified 8 individuals resilient to severe Mendelian childhood diseases [40]. These individuals had known pathogenic mutations for highly penetrant diseases, yet had not reported clinical manifestations of the disease. They postulate that protective genetic variants may be identifiable that are acting to attenuate the expression of disease in these individuals.

Various combinations of germline cancer susceptibility mutations most likely interact in unique ways. Some combinations may potentiate risk for specific malignancies while others may attenuate risk, perhaps depending on specific pathway interactions. This is likely to create difficulty in ascribing a particular cancer syndrome to a family or individual. As genetic testing becomes more widely available and used, it will be critical to expand our knowledge of the interactions among germline mutations and the ultimate genotype-phenotype consequences.

As costs of MGPT decrease, the risk of missing pathogenic mutations may outweigh the arguments against panel testing. MGPT offers lower cost of testing per gene [41]. However, the summative costs of genetic testing should take in to account the cost of screening and heightened surveillance for each positive result. On a societal level, the added costs of screening will need to be evaluated in the context of earlier diagnosis and treatment of the associated malignancies. For the individual, while insurance generally covers the costs of National Comprehensive Cancer Network (NCCN) guideline-supported risk management for known pathogenic mutations, costs may be of particular concern to uninsured or underinsured patients. Furthermore, discussion of patient concerns regarding genetic privacy and federal and state laws is needed.

While our understanding of cancer heritability, and particularly the interplay among multiple pathogenic mutations, remains unclear, it is important to acknowledge and accept the uncertainty while striving to advance the field toward a more comprehensive understanding. Prior to initiating testing, it is important to ensure that both the counselor and the patient are comfortable with results of unknown significance [16]. Ideally, physicians and counselors collaborate with patients throughout the process to explain fully the known and yet unknown before working with them to make informed, individualized plans for ongoing care and surveillance based on the profile of cancers that have been reported in association with the mutations. With time, ongoing clarifications of genotype-phenotype-environment relationships will result in adjustments to current clinical screening guidelines and allow for more accurate personalized surveillance.

### Screening our patients

While our understanding of the interplay among combinations of germline cancer susceptibility gene mutations remains incomplete, we need to be prepared to provide counseling and screening recommendations to the best of our abilities. Available screening guidelines for each relevant mutation should be reviewed (Table 2), however, it is critical that we individualize care to each patient. A review of consensus guidelines, emerging guidelines and a patient’s family history of cancer should all be considered when working with patients to make informed and shared decisions on screening.

Management of patients with “incidentally” discovered mutations can be particularly challenging, especially when established guidelines call for radical procedures (e.g. total gastrectomy in CDH1) in patients and families without a classic clinical history concerning for that cancer predisposition syndrome. For this reason, current practice is evolving to rely on clinical concordance with personal and family history in making specific screening or therapeutic decisions. In the case that genetic test results reveal a pathogenic or likely pathogenic mutation in an individual without a personal or family history suggestive of that particular cancer pre-disposition syndrome, less rigid screening protocols could be considered. For example, if a patient tests positive for a pathogenic mutation in MSH6 but does not have a personal or family history suggestive of classic Lynch Syndrome, delay of initial colonoscopy to age 25–30 or less frequent colonoscopies could be considered. Cascade testing may also be discouraged in extended family.

In the case that an individual has a family history of cancers that lie outside of the established spectrum of cancer predisposition identified by germline mutations, the 10-year rule may be utilized. Screening for that particular malignancy may be instituted when the individual is 10 years younger than the earliest known diagnosis in the family. This 10-year rule may be applied to first- and second-degree relatives.

#### Patient 1

**Fig. 1:**
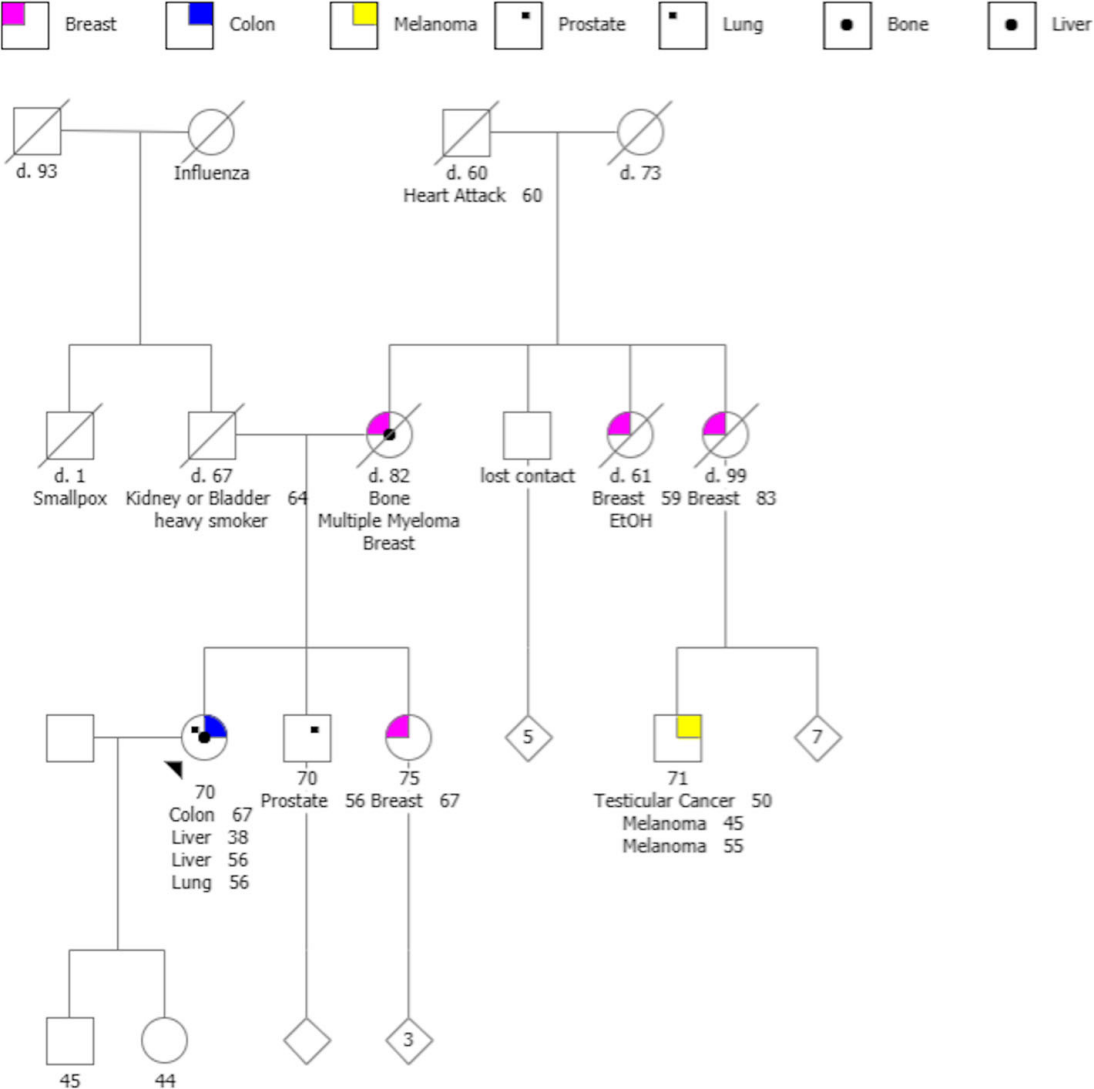
Pedigree for Family 1**.** The arrow indicates Patient 1, the proband and index case for Family 1. Her genetic testing revealed: BAP 1: Exon 12 c.1185dupA (p.Asp396Argfs*2), pathogenic MSH6: Intron 9 c.4002-2A > G (splice acceptor), likely pathogenic. RECQL4: Exon 14 c.2296C > T (p.Gln757*), pathogenic (heterozygous). All other family members declined genetic testing

MGPT was performed after her colon cancer had negative IHC staining for *MSH6*, and confirmed a germline mutation in *MSH6*, while revealing additional mutations in *BAP1* and *RECQL4* genes (Table 1, Fig. 1). Although she had an extensive personal history of cancer suggestive of cancer predisposition, her clinical phenotype and family history would not have predicted either *BAP1* or *RECQL4* mutations prior to testing.

Her screening regimen consists of a combination of the recommendations for BAP1 Tumor Predisposition Syndrome and Lynch Syndrome, respectively. She is seen by dermatology every 6 months for full body skin examination (risk for melanoma and basal cell skin cancer with *BAP1*), ophthalmology every 6 months for dilated fundus examination and annually for ultrasound biomicroscopy (UBM) (risk for uveal melanoma with *BAP1*). She also undergoes annual renal ultrasound, and MRI chest and abdomen every 2 years (risk for internal malignancies associated with *BAP1*, including mesothelioma and renal cell carcinoma) [13]. She continues annual colonoscopy and mammogram (risk for colon and breast cancer in Lynch Syndrome). She had a hysterectomy prior to her diagnosis of Lynch syndrome, but pelvic ultrasound and CA-125 are monitored annually (risk for ovarian carcinoma). UV-minimization has been emphasized given her history of both *BAP1* and *RECQL4* mutations. No specific screening for osteosarcoma is performed.

#### Patient 2

**Fig. 2:**
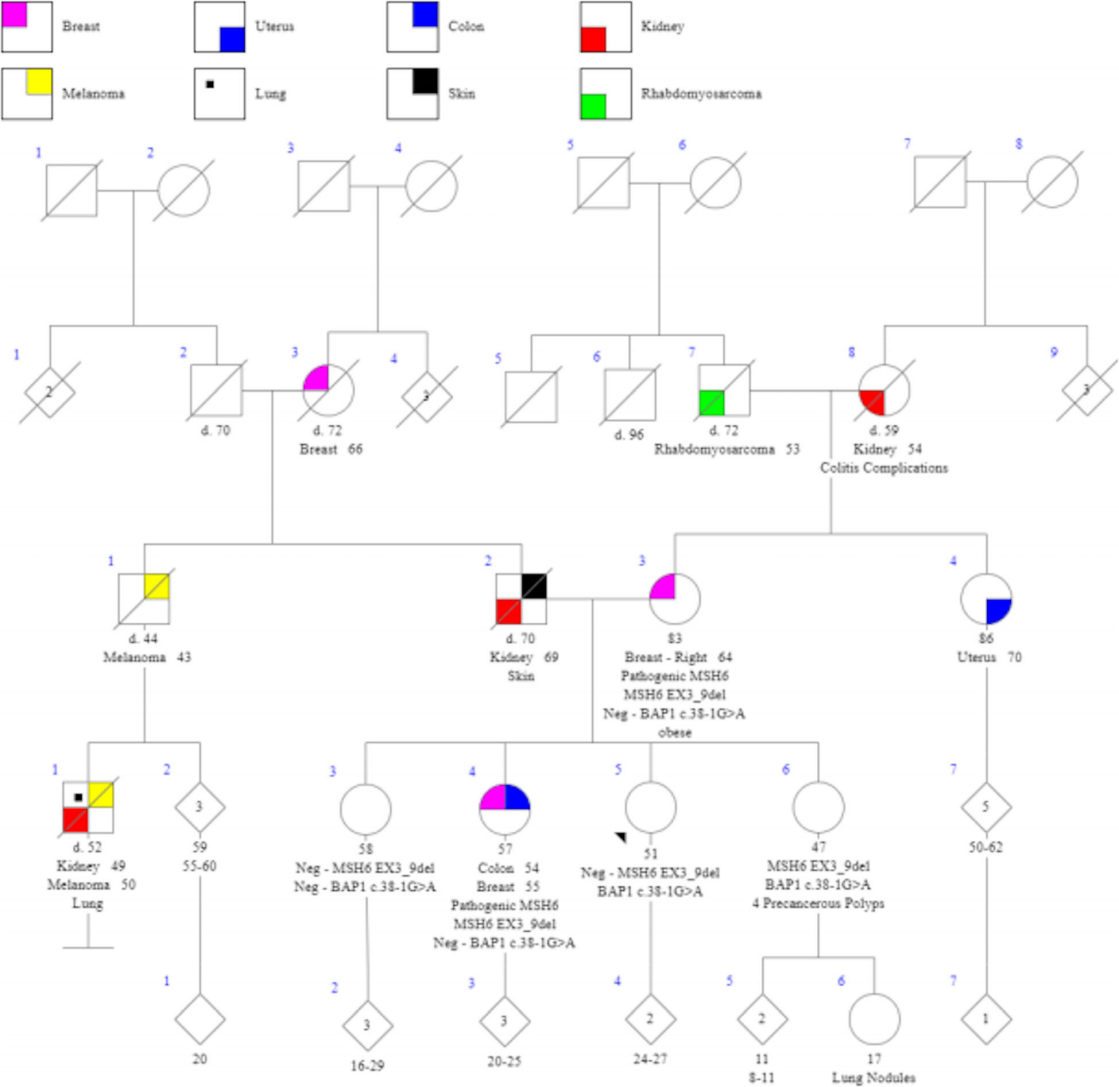
Pedigree for Family 2

The sisters in Family 2 demonstrate a Mendelian inheritance pattern of the two familial cancer predisposition mutations in *MSH6* and *BAP1* (Table 1, Fig. 2)*.* One sister (IV-3) did not inherit either mutation; one (IV-4) inherited just the MSH6 mutation, one (IV-5, the proband in this study) inherited just the *BAP1* mutation, and one (IV-6) inherited both *MSH6* and *BAP1* mutations.

The arrow indicates Patient 2, the proband for Family 1. The sisters in generation IV of Family 2 demonstrate a Mendelian inheritance pattern of the two familial cancer predisposition mutations in *MSH6* and *BAP1* (MSH6: Ex3_9del, pathogenic; BAP1: c.38-1G > A, likely pathogenic)*.* One sister (IV-3) did not inherit either mutation; one (IV-4) inherited just the MSH6 mutation (index case for Family 1), one (IV-5, the proband in this study) inherited just the *BAP1* mutation, and one (IV-6) inherited both *MSH6* and *BAP1* mutations.

Our patient was found to have a *BAP1* c.38-1G > A likely pathogenic variant and site-specific testing for the familial *MSH6* mutation was negative. She is screened with full body skin examinations every 6 months, dilated fundus examinations every 6 months with annual UBM, annual renal ultrasound and MRI chest and abdomen every 2 years.

The index case (IV-4) had a history of a tubular adenoma in the hepatic flexure, several small adenomas, a hyperplastic polyp, and two tubular adenomas in the ascending colon and sigmoid colon at age 51; then a tubular adenoma in the sigmoid colon and a right hepatic mass with no definitive invasive carcinoma at 54. She underwent a prophylactic right hemicolectomy a month later that revealed a 2.6-cm moderately differentiated mucinous adenocarcinoma, with invasion through the muscularis propria and superficially into subserosa adipose tissue (Stage 2). Loss of nuclear expression of *MSH6* suggested a high probability of Lynch syndrome, in the absence of a previous family history of colorectal cancer. Ambry Genetics panel testing revealed an *MSH6* EX3-9del. She subsequently underwent a prophylactic total abdominal hysterectomy with bilateral salpingo-oophorectomy (TAHBSO), which revealed a Stage IA, grade 1 endometrial cancer at 54. At 55, she was diagnosed with a Stage 1B ER/PR-negative left-sided breast cancer, following routine mammography. Immunohistochemical studies performed on the breast biopsy tissue demonstrated intact expression of DNA mismatch repair proteins MLH1, MSH2, MSH6 and PMS2, suggesting that her breast cancer was sporadic and not associated with microsatellite instability.

The youngest sister (IV-6) was found to have both familial mutations after testing for a broad profile (Supplemental Table 2). She was noted to have a personal history of 4 precancerous colorectal polyps at the time of her genetics visit. She has decided to undergo a combination of both screening regimens but has yet to manifest a malignancy at age 47.

The eldest sister (IV-3) tested negative for both familial mutations through site-specific testing for the familial variants through Ambry Genetics.

## Conclusions

Cancer genetics is advancing quickly as a tool for precision medicine as we move into an era of whole-exome and whole-genome sequencing. With large panel testing becoming more widely available and affordable, the challenge of counseling and managing patients with mutations in multiple germline cancer susceptibility genes, with incompletely understood interactions, will become more prevalent. To address our questions and uncertainties, we must be prepared to track, research and share our insights into the human genome. As we work to advance the field toward a more comprehensive understanding of predictive cancer genetics and the complex interplay of other genomic and environmental exposure effects, it is important that we work to individualize care for each patient while recommending a rational plan based on existing data.

We support broad MGPT in assessing patients and families for inherited predisposition to cancer and other diseases. The practice of clinical genetics is in a period of rapidly advancing understanding of genotype-phenotype correlations. While this raises puzzling and challenging questions, only through more detailed, open-ended testing and meticulous data collection will we better tease apart the complex results. Ironically, this conundrum highlights the continued relevance and importance of family history as a practical guide, despite the immense and improving technology at our disposal.

## Supplementary Information

**Additional file 1.** Invitae Multi-Cancer Panel. Broad Profile Testing for Patient 2’s youngest sister (IV-6).

## Data Availability

The datasets used and/or analyzed during the current study are available from the corresponding author on reasonable request.

## Abbreviations

MGPT: Multi-gene panel testing
NGS: Next-generation sequencing
VUS: Variants of unknown significance
BAP-1: BRCA-1 associated protein 1
MSH6: MutS Homolog 6
ACMG-AMP: American College of Medical Genetics and Genomics-Association for Molecular Pathology
AST: Atypical Spitz tumors
MM: Malignant mesothelioma
CM: Cutaneous melanoma
BCC: Basal cell carcinoma
RTS: Rothmund-Thompson Syndrome
BGS: Baller-Gerold Syndrome
CDKN2A: Cyclin-dependent kinase inhibitor
MC1R: Melanocortin-1 receptor
MRI: Magnetic resonance imaging
IHC: Immunohistochemistry
NCNN: National Comprehensive Cancer Network
UBM: Ultrasound biomicroscopy
TAHBSO: Total abdominal hysterectomy with bilateral salpingo-oophorectomy
