# Supplementary material for: Clinical challenges in interpreting multiple pathogenic mutations in single patients
Source: Hered Cancer Clin Pract. 2021 Feb 4;19:15. doi: 10.1186/s13053-021-00172-3 (PMC7863461; doi:10.1186/s13053-021-00172-3)
Supplement: Supplementary file 1 — Additional file 1. Invitae Multi-Cancer Panel. Broad Profile Testing for Patient 2’s youngest sister (IV-6). [file 13053_2021_172_MOESM1_ESM.docx]

**Supplemental Data**

**Supplement 1:** InVitae Multi-Cancer Panel

The following 80 transcripts were used in analysis for Patient 1:

ALK (NM_004304.4), APC (NM_000038.5), ATM (NM_000051.3), AXIN2 (NM_004655.3), BAP1 (NM_004656.3), BARD1 (NM_000465.3), BLM (NM_000057.3), BMPR1A (NM_004329.2), BRCA1 (NM_007294.3), BRCA2 (NM_000059.3), BRIP1 (NM_032043.2), CASR (NM_000388.3), CDC73 (NM_024529.4), CDH1 (NM_004360.3), CDK4 (NM_000075.3), CDKN1B (NM_004064.4), CDKN1C (NM_000076.2), CDKN2A (NM_000077.4), CEBPA (NM_004364.4), CHEK2 (NM_007194.3), DICER1 (NM_177438.2), DIS3L2 (NM_152383.4), EGFR (NM_005228.3: c.2369C>T p.Thr790Met variant only), EPCAM (NM_002354.2: Deletion/duplication testing only), FH (NM_000143.3), FLCN (NM_144997.5), GATA2 (NM_032638.4), GPC3 (NM_004484.3), GREM1 (NM_013372.6: Promoter region deletion/duplication testing only), HOXB13 (NM_006361.5: c.251G>A p.Gly84Glu variant only), HRAS (NM_005343.2), KIT (NM_000222.2), MAX (NM_002382.4), MEN1 (NM_130799.2), MET (NM_001127500.1), MITF (NM_000248.3: c.952G>A p.Glu318Lys variant only), MLH1 (NM_000249.3), MSH2 (NM_000251.2), MSH6 (NM_000179.2), MUTYH (NM_001128425.1), NBN (NM_002485.4), NF1 (NM_000267.3), NF2 (NM_000268.3), PALB2 (NM_024675.3), PDGFRA (NM_006206.4), PHOX2B (NM_003924.3), PMS2 (NM_000535.5), POLD1 (NM_002691.3), POLE (NM_006231.3), POT1 (NM_015450.2), PRKAR1A (NM_002734.4), PTCH1 (NM_000264.3), PTEN (NM_000314.4), RAD50 (NM_005732.3), RAD51C (NM_058216.2), RAD51D (NM_002878.3), RB1 (NM_000321.2), RECQL4 (NM_004260.3), RET (NM_020975.4), RUNX1 (NM_001754.4), SDHA (NM_004168.3), SDHAF2 (NM_017841.2), SDHB (NM_003000.2), SDHC (NM_003001.3), SDHD (NM_003002.3), SMAD4 (NM_005359.5), SMARCA4 (NM_001128849.1), SMARCB1 (NM_003073.3), SMARCE1 (NM_003079.4), STK11 (NM_000455.4), SUFU (NM_016169.3), TERC (NR_001566.1), TERT (NM_198253.2), TMEM127 (NM_017849.3), TP53 (NM_000546.5), TSC1 (NM_000368.4), TSC2 (NM_000548.3), VHL (NM_000551.3), WRN (NM_000553.4), WT1 (NM_024426.4)

**Supplement 2:** Broad Profile Testing for Patient 2’s youngest sister (IV-6)

The following 67 transcripts were used in analysis:

*AIP, ALK, APC, ATM, BAP1, BARD1, BLM, BRCA1, BRCA2, BRIP1, BMPR1A, CDH1, CDK4, CDKN1B, CDKN2A, CHEK2, DICER1, EPCAM, FANCC, FH, FLCN, GALNT12, GREM1, HOXB13, MAX, MEN1, MET, MITF, MLH1, MRE11A, MSH2, MSH6, MUTYH, NBN, NF1, NF2, PALB2, PHOX2B, PMS2, POLD1, POLE, POT1, PRKAR1A, PTCH1, PTEN, RAD50, RAD51C, RAD51D, RB1, RET, SDHA, SDHAF2, SDHB, SDHC, SDHD, SMAD4, SMARCA4, SMARCB1, SMARCE1, STK11, SUFU, TMEM127, TP53, TSC1, TSC2, VHL, XRCC2*
